# A qualitative study of the factors that influence mothers when choosing drinks for their young children

**DOI:** 10.1186/1756-0500-7-430

**Published:** 2014-07-05

**Authors:** Alexandria Hoare, Monica Virgo-Milton, Rachel Boak, Lisa Gold, Elizabeth Waters, Mark Gussy, Hanny Calache, Michael Smith, Andrea M de Silva

**Affiliations:** 1 Jack Brockhoff Child Health & Wellbeing Program. McCaughey VicHealth Centre for Community Wellbeing. Melbourne School of Population & Global Health, The University of Melbourne, Melbourne, Australia; 2 Deakin Health Economics. Deakin Population Health SRC, Faculty of Health, Deakin University, Melbourne, Australia; 3 Department of Dentistry and Oral Health, La Trobe Rural Health School. La Trobe University, Melbourne, Australia; 4 Dental Health Services Victoria, 720 Swanston St, Carlton, VIC 3053, Australia; 5 Oral Health Service, Barwon Health, Melbourne, Australia; 6 Melbourne Dental School, University of Melbourne, Melbourne, Australia

## Abstract

**Background:**

The consumption of sweetened beverages is a known common risk factor for the development of obesity and dental caries in children and children consume sweet drinks frequently and in large volumes from an early age. The aim of this study was to examine factors that influence mothers when choosing drinks for their children.

**Method:**

Semi-structured interviews (n = 32) were conducted with a purposive sample of mothers of young children from Victoria’s Barwon South Western Region (selected from a larger cohort study to include families consuming different types of water, and different socioeconomic status and size). Inductive thematic analysis was conducted on transcribed interviews.

**Results:**

Several themes emerged as influencing child drink choice. Child age: Water was the main beverage for the youngest child however it was seen as more acceptable to give older children sweetened beverages. Child preference and temperament: influencing when and if sweet drinks were given; Family influences such as grandparents increased children’s consumption of sweet drinks, often providing children drinks such as fruit juice and soft drinks regardless of maternal disapproval. The Setting: children were more likely to be offered sweetened drinks either as a reward or treat for good behaviour or when out shopping, out for dinner or at parties.

**Conclusions:**

Limiting intake of sweet drinks is considered an important step for child general and oral health. However, the choice of drinks for children has influences from social, environmental and behavioural domains, indicating that a multi-strategy approach is required to bring about this change.

## Background

The consumption of sweetened beverages is a known common risk factor for the development of obesity and dental caries in children. Dental caries and obesity are prevalent Australian childhood conditions, affecting about one third of children of preschool age [1,2]. Both conditions can lead to developmental problems, adverse education and social impacts, lowered quality of life and poor health outcomes tracking through to adulthood [3-7]. The greatest burden is experienced by those of lower socioeconomic background who face an increased risk of both dental caries and obesity [2,8]. A common risk factor for these conditions is a diet high in refined carbohydrates particularly sugars, led by the consumption of soft drinks and other sweetened beverages [8-13].

There is a demonstrable benefit on dental health if water is consumed in preference to sweetened beverages and numerous studies have reported the benefits of fluoridated water on children’s dentition [14,15]. An Australian study reported that lifetime exposure to fluoridated water significantly reduced the risk of caries in children’s deciduous teeth (up to 55% fewer surfaces affected) and permanent teeth (up to 65% fewer surfaces affected) compared to children without exposure [16]. Furthermore, water fluoridation can benefit at-risk children as it has been associated with fewer childhood caries regardless of socio economic background [17,18]. However, water fluoridation will not have an impact on children’s dental outcomes if fluoridated tap water is not being consumed.

In 2005, the Victorian Child Health and Wellbeing Survey (VCHWS) included a question to capture tap water consumption for the first time, with findings demonstrating that 50% of children residing in rural and regional Victoria were not drinking tap water at the time [19]. Population trends towards consumption of bottled waters (low or no fluoride) or a preference for fruit juice, cordial and soft drinks that contain high levels of sugars and acid can be related to patterns of consumption of tap water [20].

Consumption of sugar sweetened beverages (SSB) (which includes soft drinks, soda, fruit drinks, fruit juice, sports drinks, sweetened iced tea, squashes, and lemonade) in pre-schoolers and school aged children remains high, with a recent systematic review showing a positive association between intakes of SSB and weight gain and obesity in children [21]. Research has identified that children consuming two or more cups of soft drink per week are 1.3 times more likely to be obese with the association greater at higher levels of consumption [22]. Alarmingly, over 13% of 2–3 year old children are consuming SSB on a daily basis [23]. Consumption of SSB is nearly twice as common in children from low SES areas with this group also having nearly double the rates of obesity [24].

Parents are responsible for their children’s diets and as such when trying to improve children’s diets it is important to understand the influences on parents in their decision making. Although limited, research suggests a relationship between a maternal health motivation and the quality of her child’s diet [25]. Factors such as health, mood, price, convenience and weight control have been shown to influence maternal food choice and these factors may also influence food selection for children [26]. A qualitative study by Malbach et al. (2009) identified that parents felt nutrition should determine their food selections but it was not always the case. They were often influenced by child preference for certain foods, maintaining harmony within the family, familiarity of brands, habitual purchases, price and marketing [27].

An earlier study conducted by Devine and Olson [28] interviewed a small group of women with young children. The women took on the role of providing food for the children and to encourage and inform them about healthy eating [28]. However, keeping the peace at mealtimes often resulted in the women ‘shutting their eyes’ or making compromises about what the children ate [28]. Similar research conducted by Patrick and Nikolas [29] concluded that convenience, time restraints, income and parental education played an important role in children’s eating patterns and diet [29].

Given that both dental caries and overweight and obesity are considered largely preventable, the increasing incidence of both conditions means that sustainable and equitable solutions to address these health issues are needed. A significant information gap has been knowledge of the drivers of beverage consumption for young children. Investigating such factors could provide a contemporary and unique contribution to integrated health, education and social policy and program directions.

## Methods

This qualitative study formed a part of a larger birth cohort study ‘Splash!’ [30] which is following a birth cohort prospectively over 5 years. The rationale and methods for the Splash! study are described in detail elsewhere [30]. The reporting of this current study is consistent with the RATs guidelines for reporting of qualitative studies.

The current study was designed to explore the determinants of parental choices concerning children’s beverage consumption, specifically the attitudes and perceptions of parents towards drink choice and the importance of perceived water quality, water fluoridation, water costs and other identified factors affecting drink choice.

A sub-sample of 32 mothers was selected from the Splash! main study sample (n = 458) to provide a range of demographics, including main source of water consumed (tank, tap, bottled), socioeconomic status (education, income) and number of children in the family. These characteristics were chosen as they are analytically important and have been shown to play an important role in individual and family oral health [31,32]. Sampling continued until there was a sufficient range of these demographic variables amongst participants [31]. Parents who had agreed to participate in an in depth interview on the original Splash! consent form were called by the researchers and invited to participate if their child taking part in the study was aged six months or older at the time of the interviews.

In-depth semi-structured interviews (n = 32) were conducted across the Barwon South Western Region of Victoria, Australia from March to June 2011. The interview guide consisted of 43 open-ended questions and each participant was asked the same questions but not necessarily in the same order. Interview questions explored participants’ attitudes and perceptions towards drink choice, water quality and oral health. Questions examined participants’ thoughts on water, how people make choices when it comes to the drinks they consume, and their beliefs around oral health. Interviews continued until no new topics or themes emerged.

Consistent with standard practice the development of the interview guide was informed by a comprehensive review of the literature to identify key areas of interest [33]. The draft interview questions were piloted with four primary caregivers not involved in the Splash! study whose infants were between 6 months and 3 years of age. This was to ensure questions were interpreted and answered to reflect true differences in primary caregivers beliefs, attitudes and perceptions towards individual, child and family oral health [33]. No changes were required to the interview guide.

Two members of the research team (one interviewer and one note taker) conducted each interview. Three interviewers conducted the interviews after being trained appropriately prior to the commencement of the interviews to ensure consistency and reliability of the interviews. The interviews were conducted in participants’ homes or at a location convenient to them and took approximately 40–50 minutes. All interviews were digitally recorded using an Olympus digital voice recorder (WS-321 M), transcribed verbatim and verified by independent researchers.

The content of the interviews was discussed amongst the interviewers and research team throughout the data collection and analysis process which shaped and informed subsequent interviews and analyses [31,34].

Ethics approval was received from the Human Research Ethics Committees of the University of Melbourne (Melbourne, Australia) and Barwon Health (Geelong, Australia). Participants attending interviews provided written and verbal consent for participation, recording of discussion and use of the data. All participant information was de-identified and all were given a participant identification number.

Transcripts were imported into NVivo 9.1 (QSR International, 2011) for coding. A thematic analytic technique was used to analyse the transcripts [31]. This method involved constantly comparing, classifying, grouping and refining sections of transcripts to generate a collection of themes within the data [31]. Specifically, items in each interview were compared with the other interview transcripts to inform the development of analytic categories and conceptualise relationships between the data [35,36].

All analyses were performed by the three interviewers as they were the most immersed in the data and brought this experience and understanding to the process of analysis [37]. Inductive thematic content analysis was conducted with each transcript. In this coding process researchers examined the data so that embedded relationships and meanings could emerge [31,38]. Using a similar process interviewers then identified common codes across participants [31]. The third phase involved looking at how codes could be linked, and in this process categories which relate to different aspects of the issues being explored were developed [37]. The final phase of analyses involved moving beyond these categories to identify themes. This process required the researchers to move beyond just describing the categories identified to generating themes which provide interpretation of the issues being explored [37].

Transcripts were cross coded to verify consistency in coding [39]. The three interviewers/coders met as a group to discuss any inconsistencies in coding and reach a consensus across all major themes. This process of repetitive checking between coder’s interpretations helped to ensure that the results of the research reflected the participants experiences and provided an accurate interpretation of the data [40].

## Results

Respondents were aged 19 to 42 and were all mothers of infants aged between 6 and 12 months at the time of the interview. For about one third of mothers interviewed (34%) the infant in the study was their first child and they only had one child living with them, 31% had two children, 22% had three children, and 13% had four children living with them (inclusive of study child). Ninety per cent of the interview participants were Australian-born and all spoke English at home. Over three quarters of the participants had completed secondary school and 34% had completed a bachelor level degree. Over half of mothers engaged in casual, full or part time work. Of the interviewed sample, 25% had a health care card (that provides reduced healthcare costs for low income households and/or people with chronic medical conditions).

In the thematic analyses, twelve major topic areas were identified for Drinks, 12 for Food and 14 for Oral health. This paper will report only on the topic areas related to Drinks. Within the 12 topic areas identified related to children’s beverage intake five main categories emerged; drink choice, fluoride, influences, consumption of sweetened drinks and introduction of water. Themes that emerged from these categories include health, age appropriateness of drinks, child’s temperament, drinks preference and social influences. Results are presented below according to main topic area, with illustrative quotes of the emerging themes.

### Drink choice

Mothers reported that water was the main beverage for their youngest child. There was consensus that water was seen as a ‘*healthy choice’* and was ‘*good for you’*. Several stated that they gave their children water to drink because it was good for their teeth, had less sugar than other beverages and was good for their health. Water was seen as hydrating and cleansing with sweetened beverages being described as unnecessary for infants by some of the mothers. Mothers provided children with tap water as it was easy and convenient with limited costs, although some chose tap water specifically for the fluoride content. When asked why they gave their children water to drink responses included;

ID 133 ‘*because of their teeth and its better for you…and its cheaper’*

ID 138 ‘*it’s a lot better for them, it’s what their body needs, it’s better for all parts of their body’*

ID 7 ‘*Coz it’s better for them and it would be better for their teeth’*

Of the few that gave their children bottled water it was for reasons concerning taste of tap water, having old pipes in the house and to limit fluoride ingestion.

Those that provided their children with a sweetened beverage would usually choose fruit juice, as it was perceived to have health benefits such as vitamins.

ID 281 *‘Probably if it was a sweet drink I would give a juice, at least . . . at least she is going to get some health benefits from having juice’*

ID 317 *‘I’d give like watered down orange juice like juices and stuff because I know that they are good for them’*.

Flavoured milk was also a popular sweetened drink for children. Again mothers felt that milk drinks would provide some health benefits such as calcium. This idea was linked with the perception that milk was necessary for growing children. Some mothers described flavoured milk as being one of the better choices when choosing sweetened beverages:

ID 92 ‘*I guess it* [*flavoured milk*] *is the lesser of most other evils. At least they get a little bit of nutrients out of it. Umm* . . *. yea*, *just I guess maybe not as much sugar*’.

Mothers that had older children felt that it was important to give their youngest children water to drink but it was okay to give the older children sweetened drinks. As children got older it was seen to be more acceptable to give them sweetened beverages.

ID 11 ‘*I guess further down the track when she*’*s the boys age I would feel ok about offering her flavoured milk and maybe a bit more juice*…’.

The main concerns and reasons for not giving children sweetened drinks such as fruit drinks, juices and soft drink were the high sugar content, they are ‘*not needed*’ for children and they are ‘*bad for teeth*’. Some made note of behavioural changes after their child consumed sweetened drinks such as hyperactivity and restlessness. As one mother noted she prefers to provide her child with water over sweetened drinks;

ID 230 ‘*for health the health benefits and to avoid umm like with the tooth decay and sugary drinks and also the hyperactivity*’.

### Fluoride

Views varied on the benefits of fluoride in tap water, although most mothers felt that fluoride was a benefit and was good for teeth. Several stated that they prefer to provide their children with tap water over bottled water because of the fluoride content. Mothers in support of fluoride thought it was good for their children’s teeth and helped prevent decay.

ID 169 ‘*the fluoride in the water*… *I*’*ve heard it*’*s good for children*’*s teeth so they*’*ll continue to drink it*’

ID 247 ‘*I*’*m happy with fluoride in the water and if we didn*’*t have it we would probably be giving her fluoride tablets I imagine*’.

Those that avoided fluoride did so because of perceived negative side effects and health problems associated with fluoridated water.

ID 100 ‘*fluoride use is obviously associated with stomach cancers and it can cause thyroid problems*’ and ‘*we try and avoid as much fluoride as is possible*’.

Mothers with this view opted to give their children bottled water without fluoride regardless of the associated costs. Some mothers knew of the benefits associated with fluoridated water but were cautious of perceived negative side effects so gave their children a mixture of fluoridated tap and non fluoridated bottled water.

ID 100 ‘*I*’*m aware that fluoride is really necessary for oral health umm so the kids have a good mixture* [of tap and bottled water] *but I don*’*t just generally use tap water*’.

In some areas where the participants lived, community water fluoridation was relatively new. When fluoride was added to the reticulated water supply some mothers reportedly compensated by giving their child non fluoridated toothpaste.

ID 100 ‘*they*’*re using non*-*fluoridated toothpaste as well so they*… *you know it*’*s*, *it*’*s balance*’

ID 272 ‘*if we*'*ve got fluoride in the water I would give the children toothpaste without the fluoride in it to balance it out because I just think it*’*s just a pretty harsh chemical if they*’*re getting lots of it*’.

### Child influences

Child’s preference and temperament, parenting style, other family members, own experiences, marketing and social settings were all said to influence the choice of beverage provided to children.

Child preference: Mothers reported that their children preferred sweetened drinks and would give sweetened drinks as a treat or because it was the child’s preferred choice.

ID 301 ‘*if they get a choice they wouldn*’*t choose water first so they would probably have you know the flavoured waters or juices or whatever as a first preference they don*’*t always get it but they do get it a fair bit*’.

When asked about the reason they gave their children cordial (fruit-based sugar syrup which is diluted before drinking) to drink one mother responded;

ID 237 ‘*Because they like it* . . . *I suppose*’.

Several mothers stated that their children had a preference for water or that the child would choose water over a sweetened drink as one mother explained;

ID 13 ‘*they*’*ve gotten to a point where they actually choose to drink water over soft drink*’.

Child temperament emerged as a major factor influencing the provision of sweetened beverages. Often sweet drinks would be given to ‘*keep the peace*’ if the child had been nagging.

ID 237 ‘[*child*] *makes a lot of noise about having to drink water so I will say*, *then have the damn cordial*’

ID 301 ‘*you do a lot of stuff for peace even though you know it*’*s not the right the best decision for them health wise*’.

Parenting style was also an important influencing factor in beverage choice. Some mothers would conform to their child’s demand and preferences where others would ignore their child’s requests for a sweet beverage. Taste was also a factor, with mothers reporting that their child disliked the taste of water, and therefore the mothers felt they had no choice but to allow the child to choose.

ID 237 ‘[*child*] *hates water*, *he has cordial and he drinks it all the time* . . .’

In contrast other mothers would not allow sweet drinks.

ID 172 ‘*I just sort of say to him no*, *no cordial now and*… *He doesn*’*t get it*’.

A proportion of older children were allowed to choose what drink they consumed, often choosing a sweetened drink. Other mothers enforced only water by restricting availability of sweetened drinks in the house.

Mothers’ own experiences were often related to their decision making. Some mothers described their own experiences about growing up and drinking cordials and fruit drinks, which has now influenced their decision to restrict the same kind of sweetened drinks from their children. One mother described the impact on her teeth from drinking ‘*sugary drinks*’ as she was growing up, as a result she now restricts them from the house

ID 172 ‘*if I don*’*t have it* [*sugary drinks*] *in the house then my kids aren*’*t going to have it and their teeth are gonna be better than mine*’.

The majority of mothers felt that they had learnt from their past and didn’t want their children to get into the same bad habits or have poor teeth like their own. Others commented that they now have the knowledge about healthy drink choice, something their own parents may not have had. On the other hand a mother discussed her childhood experience of growing up drinking cordial which has now influenced her decision to also give cordial to her children. Much of the discussion around childhood experiences involved the mothers growing up drinking water which has now been passed onto their own children.

ID 169 ‘…*I still drink the same amount of water as I do now like that was definitely pumped into us to have water and I*'*ve passed that on to my kids*’.

Family influence played a role in what the children drank. Grandparents were often identified as providing children with sweetened drinks such as fruit juice and soft drinks ‘ID 247 *she probably has juice at her grandparents*’ *house actually*’. One mother explained that she didn’t agree with the grandmother giving her children sweetened drinks but felt like it wasn’t her place to argue;

ID 301 ‘*you just think well they have had 4 or 5 kids or whatever they*’*ve had they should know*, *but I don*’*t know*…*I just respect*, *I don*’*t tell them*, *I don*’*t pull rank*’.

Another mother explained the influence of grandparents quoting

ID 272 ‘*Well she*’*s got a very loving grandparent* …*who thinks it*’*s acceptable to give 4 year olds 600 mls of soft drink*’.

Mothers indicated that sweetened beverages were consumed by children away from the home environment and social settings were one of the main reasons mothers offered their children sweetened drinks. Children would get sweet beverages as a treat if they had been out shopping for the day, went out for dinner or at parties.

ID 7 ‘*it sorta depends more on her social*… *like if they*’*re around other kids that are having soft drink she*’*ll have more soft drink*.’

Mothers felt that is was okay to provide children with sweetened beverages on these occasions as it was viewed as a treat.

ID 92 ‘*it*’*s the part and parcel of the party*, *the nice coloured blue drinks*’

ID 13 ‘*I allow them to have it*, *you know at parties*, *or special occasions or something*’.

Marketing also influenced mother’s decisions about what drinks to provide their child especially while shopping for groceries and when out shopping in general with their child. Many noted that that they thought it would be beneficial if bottled water was aimed at children with colourful packaging and smaller bottle sizes. When shopping some went on to say that if water was marketed at children it would persuade them to purchase it over fruit boxes and juices.

ID 252 ‘*even if they put water in fruit boxes there would be a huge market*’.

### Consumption of sweetened drinks

To prevent their children drinking sweetened beverages mothers thought it was important to encourage healthy choices and establish positive behaviours early in childhood. Mothers tended to agree that providing only water at a young age would mean that they would adopt that same habit later in life. Several comments were also made about not wanting children to form the bad habit of consuming sweetened drinks when they were young.

ID 138 ‘*Just the artificial rubbish in it* [*cordial*] *and the high sugar content yea I*’*m not horrible I*’*m not going to get him into the habit*.’

It was also noted on many occasions that not having sweetened drinks in the house prevented themselves and their children from consuming them. Mothers limited the amount of sweetened drinks in the house so the children only had the option of healthier beverages such as water. However a few mothers mentioned that they did have sweetened drinks in the house that were only allowed to be consumed by themselves or their partners.

ID 299 ‘*a bottle of cordial or something in the pantry which is only for daddy and they know that*’*s only for daddy*’.

In contrast to this was role modelling behaviour. Role modelling was a key way many mothers promoted water consumption, especially when children liked to drink the same beverage as the parents.

ID 169 ‘*the kids always steal my drinks so I*’*d rather them drink water than soft drink*’.

### Introduction of water

The age of introduction of water to infants varied, with ages ranging from birth until 12 months. Most mothers introduced water to their infant at around 6 months of age, and a variety of reasons were identified, including hydration, to aid digestion of solids and to avoid constipation:

ID 231 ‘*I think when she started to have solid foods so about 6 months*’

ID 230 ‘*as she started to eat more I thought well I*’*m pretty sure they have to start drinking water to avoid constipation*’.

Persistence was the most common strategy to use if the infant didn’t take the water when introduced to it. In the mother’s opinion if their infant didn’t drink the water straight away it was due to it being a different texture or temperature to their usual drink of milk or that they didn’t like the taste. Another strategy was to sweeten the water with cordial or juice if the child didn’t drink it:

ID 317 ‘*I would probably give her some juice like watered down juice just a drop of juice in there just to see if she*’*d like it*’.

Offering alternative drinks was another option, including offering different types of water such as boiled, tap, bottled or offering milk instead.

The child’s development and motor skills also impacted on the timing of introduction of water. Some children would only drink water from cups or bottles while others were spoon fed the water.

ID 247 ‘*it was more of mode of delivery issue than the flavour or anything*’

Infants born in summer were introduced to water as early as birth for hydration or if the infant was thirsty between feeds.

## Discussion

This study has identified a number of influences on maternal choices for child beverage consumption. Consistent themes emerged that may impact on the development and implementation of strategies aimed at limiting sweetened beverage consumption. It was found that most mothers felt it was best to give their child water but factors such as the taste preferences, age and temperament of the child, family influences and social settings influenced the mother’s choice of drink for their child.

This study has found the majority of mothers believe that water is the best beverage to provide for their young children. However, most mothers also perceived that their children prefer sweetened beverages, and as such they would be acting against their children’s wishes in providing water, particularly as children got older. It became harder for mothers to continue to mainly provide water and they became more lenient towards giving sweetened beverages. This is consistent with findings from Skinner et al. [41] which found that carbonated beverage consumption more than doubled in toddlers between the ages of 15 to 24 months of age. This is a critical stage when children are developing primary teeth and increasing their consumption of sweetened beverages can impact oral health. Health promotion strategies which highlight the importance of water for children of all ages are needed. .

Overweight and obesity is another concern for young children, affecting an estimated 12% of preschool children in developed countries [42]. Previous research indicates a positive association between greater intakes of sugar sweetened beverages and weight gain and obesity in children [21]. In the current study while mothers recognised that sweetened drinks were bad for teeth and caused decay, no comments were made about sweetened drinks and weight gain. Mothers may not associate the two or may believe weight gain is not an issue for their young child. Highlighting to parents the negative consequences of consuming sweetened beverages on child health in relation to both unhealthy weight gain and tooth decay seems to be required. In addition, the mixed marketing messages that parents receive in relation to what are healthy and unhealthy beverages most likely creates confusion and needs to be addressed.

In general, mothers identified the dental health benefits of fluoride, although a small number believed fluoride to be harmful. Numerous longitudinal studies have shown the benefits of fluoride with no evidence that fluoridated water contributes to detrimental health effects [16,43,44].

Children’s preference for sweetened drinks was another reason mothers identified for beverage choice. Research conducted by Beauchamp & Moran (1982) revealed that experience with sweetened water during infancy heightened the desire for sweetened beverages at the age of two [45]. As child preference was a main factor influencing mothers’ decisions, preventing consumption of sweetened drinks early in life is an important strategy to reducing children’s preference and desire for them.

Additionally, family influence played an important role in the children’s beverage consumption. This is consistent with previous research by Blinkhorn et al. which concluded that children had a considerable amount of influence in obtaining sweet drinks and food from parents and grandparents [46]. Interventions and health promotion strategies related to healthy drink choice need to target at both parents and other family members, such as older siblings and grandparents as they are important role models and food providers.

Finally, the finding that the majority of sweetened beverages were consumed outside of the household environment suggests opportunities to make water more accessible, easily available and more appealing for children. As suggested by some mothers, marketing of water to children with appealing packaging and commercial advertising may be a strategy worth testing. Interestingly, this finding contradicts the results that were published in the 2007 National Children’s Nutrition and Physical Activity Survey which reports that the majority of SSB’s consumed by children aged 2–16 years are done so in the home environment [24]. A possible reason for this discrepancy may be the younger age of the children in the current study. Mothers indicated that sweetened beverages were consumed by children away from the home as a treat or a reward for good behaviour. As children get older they may not need such incentives to maintain good behaviour. Mothers also mentioned that they were more lenient towards drink choice as children got older and were more relaxed about having SSBs in the home and allowing their older children to drink them.

This study has a number of limitations. In depth interviews are well suited to explore the perceptions and beliefs of participants concerning complex and sometimes sensitive issues whilst allowing the researcher to probe if a more detailed understanding or clarification of response is required [33,47,48]. However this approach prevents generalizations of findings to the entire population. Three interviewers were also used in this study which may have resulted in differences in style, manner and language between the interviewers potentially influencing the data collected. To minimise this a semi structured interview guide was used and all interview transcripts were cross coded to verify consistency.

## Conclusions

Population-level strategies for reducing the consumption of sweet beverages by children have the potential to reduce the prevalence of both childhood obesity and dental disease. However to be effective these strategies need to address the range of influences on parental choices for their children.

## Competing interests

The authors declare that they have no competing interests.

## Authors’ contributions

All authors (AH, MVM, RB, LG, EW, MG, HC, MS, AdS) contributed to the concept and design of the study. AH, MVM and RB collected the data, transcribed interviews and analysed the data. AH, MVM and AdS drafted the manuscript. RB, LG, EW, MG, HC and MS provided critical input into interpretation of results and re-drafting of the manuscript. All authors read and approved the final manuscript.
